# PKD2 and RSK1 Regulate Integrin β4 Phosphorylation at Threonine 1736

**DOI:** 10.1371/journal.pone.0143357

**Published:** 2015-11-18

**Authors:** Lisa te Molder, Arnoud Sonnenberg

**Affiliations:** The Division of Cell Biology, The Netherlands Cancer Inst., Plesmanlaan 121, 1066 CX Amsterdam, The Netherlands; Hungarian Academy of Sciences, HUNGARY

## Abstract

The integrin α6β4, a major component of hemidesmosomes (HDs), stabilizes keratinocyte cell adhesion to the epidermal basement membrane through binding to the cytoskeletal linker protein plectin and association with keratin filaments. Disruption of the α6β4-plectin interaction through phosphorylation of the β4 subunit results in a reduction in adhesive strength of keratinocytes to laminin-332 and the dissolution of HDs. Previously, we have demonstrated that phosphorylation of T1736 in the C-terminal end of the β4 cytoplasmic domain disrupts the interaction of β4 with the plakin domain of plectin. Furthermore, we showed that β4-T1736 can be phosphorylated by PKD1 *in vitro*, and although both PMA and EGF induced T1736 phosphorylation, only PMA was able to activate PKD1. Here, we show that depletion of [Ca^2+^]*i* augments PMA- and EGF-induced phosphorylation of β4-T1736 and that this is caused by inhibition of the calcium-sensitive protein phosphatase calcineurin and augmentation of ERK1/2 activation. We also show that in keratinocytes the PMA-stimulated phosphorylation of β4-T1736 primarily is mediated by PKD2 activation downstream of PKCδ. On the other hand, both the EGF-stimulated phosphorylation of T1736 and the EGF-induced dissolution of HDs are dependent on a functional MAPK signaling pathway, and treatment with the RSK inhibitor BI-D1870 prevented EGF-stimulated phosphorylation of β4-T1736. Moreover, phosphorylation of β4-T1736 is enhanced by overexpression of wild-type RSK1, while it is reduced by the expression of kinase-inactive RSK1 or by siRNA-mediated depletion of RSK1. In summary, our data indicate that different stimuli can lead to the phosphorylation of β4-T1736 by either PKD2 or RSK1.

## Introduction

Hemidesmosomes (HDs) are junctional protein complexes that are crucial for maintaining firm adhesion of keratinocytes to the underlying basement membrane and thus for epithelial tissue integrity [1,2]. The integrin α6β4 is an important component of HDs. Together with Bullous Pemphigoid 180 (BP180), a transmembrane collagen, it binds to laminin-332 in the epidermal basement membrane [2–4]. At the cytoplasmic face, these proteins are connected via plectin and BP230, two members of the plakin family of cytoskeletal linker proteins, to keratin intermediate filaments [5–7].

Studies with cultured keratinocytes revealed that the formation of HDs is critically dependent on the binding of plectin to the cytoplasmic domain of β4 [8–10]. Mutations that prevent this interaction lead to a loss of HDs, similarly to that seen for mutations that cause the complete absence of α6β4 or plectin [11–14]. Subsequent studies therefore have focused on the regulation of this interaction by phosphorylation as a means to promote HD disassembly in migrating keratinocytes during wound healing. It was thus demonstrated that the β4 cytoplasmic domain harbors several residues that are phosphorylated in response to stimulation of keratinocytes with agents known to cause HD disassembly, e.g. phorbol 12-myristate 13-acetate (PMA) and Epidermal Growth factor (EGF) [15–18]. Two of these residues (S1356 and S1364) are present in the connecting segment that separates the two pairs of type III fibronectin (FnIII) domains in the cytoplasmic domain of β4, while a third one (T1736) is located in the C-tail that follows the last FnIII domain. Phosphorylation of S1356 and S1364 prevents interaction of β4 with the actin-binding domain (ABD) of plectin, possibly by a mechanism of phosphorylation-dependent auto-inhibition, while T1736 phosphorylation results in the disruption of the binding site for the plakin domain of plectin that follows the ABD [16–18]. The phosphorylation of a fourth site (S1424) on β4, which is enriched in the trailing edge of migrating keratinocytes, has been associated with the dissociation of BP180 from HDs [19].

Both PMA and EGF activate the mitogen-activated protein kinase (MAPK) signaling pathway and evidence has been presented that S1356 and S1364 are phosphorylated by two components of this signaling pathway, ERK-1/2 and their downstream effector kinases RSK-1/2, respectively [17]. Recently, we have shown that PKD1, a downstream effector kinase of PKC, phosphorylates β4 at T1736 *in vitro* and that this kinase is activated in keratinocytes upon PMA stimulation [18]. However, evidence that this occurs in keratinocytes treated with PMA has not yet been reported. Furthermore, because PKD1 is not activated in keratinocytes stimulated with EGF, it is not clear which kinase is responsible for the phosphorylation of this residue downstream of EGFR activation.

Here we set out to identify the kinases that phosphorylate β4 at T1736 upon PMA and EGF treatment of keratinocytes. We show that PKD2 is robustly activated by PMA and that PKD2 phosphorylates T1736, while RSK1 is the kinase that phosphorylates this residue downstream of the EGF receptor in keratinocytes. Furthermore, we show that phosphorylation of T1736 is influenced by cytoplasmic calcium levels, which is in part regulated by the serine-threonine phosphatase calcineurin.

## Materials and Methods

### Antibodies

The generation of the rabbit polyclonal antibodies specific for phosphorylated β4 (T1736, S1364 or S1356) and the rabbit polyclonal antibodies recognizing the first pair of FNIII domains (residues 1115–1355) of β4 have been previously described [17, 18, 20]. The rabbit polyclonal antibodies against PKD1 (also reacting with PKD2), PKD3 (D57E6), phospho-PKD1 (S916), which crossreacts with phospho-PKD2, phospho-PKD (S744/748) recognizing the phosphorylated activation loop in PKD1/2/3, phospho-pan PKC (βII S660), RSK2 (D21B2), phospho-p90RSK (T359/S363), phospho-p90RSK (S380) and phospho-Akt (S473) and the rabbit monoclonal antibody (mAb) against phospho-p44/42 ERK1/2 (T202/Y204) were purchased from Cell Signaling. Antibodies against PKCδ (sc-937) and PKCε (sc-214) were from Santa Cruz Biotechnology. The rat mAb against integrin β4 (439-9B) and the mouse mAb against plectin (31/plectin) were from BD Bioscience. The mouse mAb against α-tubulin (clone B-5-1-2) was obtained from Sigma-Aldrich. Anti-mouse and anti-rabbit HRP-conjugated secondary antibodies were purchased from GE Healthcare, and TexasRed-conjugated goat anti-mouse and FITC-conjugated goat anti-rat were from Invitrogen.

### Cell culture

PA-JEB immortalized keratinocytes were isolated from a patient with Pyloric Atresia associated with Junctional Epidermolysis Bullosa (PA-JEB) [8]. Since the derivation of the cell line was done for diagnostic purposes, the research using these cells was exempt of the requirement for ethical approval. PA-JEB/β4 keratinocytes stably expressing wild-type β4 were generated by retroviral transduction and maintained in serum-free keratinocyte medium (KGM; Invitrogen) supplemented with 50 μg/ml bovine pituitary gland extract, 5 ng/ml EGF, and antibiotics (100 units/ml streptomycin and 100 units/ml penicillin), as described previously [20]. A431 epidermoid carcinoma cells, obtained from the American Type Culture Collection (Manassas, VA), were cultured in Dulbecco’s modified Eagle’s medium (DMEM) containing 10% heat-inactivated fetal calf serum (FCS), and antibiotics [21].

### Immunofluorescence

PA-JEB/β4 cells were seeded on glass coverslips and cultured in either low (KGM; 0.09 mM Ca^2+^) or high calcium (DMEM; 1.8 mM Ca^2+^) medium for 24h, or cultured overnight in KGM medium deprived of growth factors, pre-treated with UO126 (10 μM; Calbiochem) for 1h and stimulated with 50 ng/ml EGF (Sigma-Aldrich) for 30 min. The cells were fixed with 1% paraformaldehyde and permeabilized with 0.2% Triton-X100 for 5 min. Cells were blocked with PBS containing 2.5% bovine serum albumin (Sigma-Aldrich) and incubated with the primary antibodies (mAb 31/Plectin (dilution 1:100; final concentration 2.5 μg/ml) and mAb 439-9B against β4 (dilution 1:100, final concentration 2 μg/ml)) for 1h. Cells were washed three times before incubation with the secondary antibodies for 1h. After three wash-steps with PBS, the coverslips were mounted onto glass slides in Mowiol-DAPCO and studied by using a Leica TCS SP5 confocal microscope.

The degree of co-localization between β4 and plectin was quantified using the Pearson’s correlation coefficient generated with the JaCoP macro, Image-J software [22]. Statistical significance was analysed using unpaired t-tests (corrected for multiple testing with the Bonferroni method) in SPSS and the graphs were made in GraphPad Prism 6.

### cDNA constructs, siRNAs and transfection

The plasmids encoding human RSK1, mouse RSK2 and their kinase inactive mutants, RSK1 (K94/447R) and RSK2 (K100/541R), were kindly provided by Dr. J. Blenis (Harvard Medical School, Boston, MA). The RSK1 (SI02223060), RSK2 (SI00288190), PKD1 (SI00301350), PKD2 (SI02224768), PKCδ (SI02660539) and PKCη (SI02224082) siRNAs were purchased from QIAGEN and the PKCε siRNA (J-004653-08) was obtained from GE Healthcare. PA-JEB/β4 keratinocytes were transiently transfected with cDNAs or siRNAs using lipofectamine^®^ 2000 (Invitrogen). Lipofectamine (14 μl/ml) and cDNA (10 μg/ml) or siRNA (0,5 μM) solutions in Opti-MEM were mixed (1:1) and incubated for 30 min at room temperature. Cells were incubated with the transfection solution for approximately 15h.

### Western blotting

For western blot analysis, (transfected) PA-JEB/β4 keratinocytes and A431 cells were deprived of growth factors overnight in growth factor-free KGM and in DMEM without FCS, respectively. Cells were pre-treated with the calcium chelator BAPTA-AM (20 μM) and/or the phosphatase inhibitors FK506 (5–100 nM; Cell Signaling) or cyclosporin A (50–250 nM; Cell Signaling), the kinase inhibitor Gӧ6983 (100 nM—1 μM; Calbiochem), UO126 (2–10 μM; Calbiochem), BI-D1870 (1–20 μM; Enzo Life Sciences), GDC-0941 (8 nM-1 μM; Selleckchem), or AZD 8055 (0.8–100 nM; Selleckchem) for 30–60 min, before they were stimulated with 50 ng/ml EGF (Sigma-Aldrich) or 100 ng/ml PMA (Sigma-Aldrich) for 10 min. For studies with the calcium ionophore A23187, PA-JEB/β4 keratinocytes were grown in complete KGM (with pituitary gland extract and EGF) and pre-treated with BAPTA-AM (20 μM) and/or A23187 (1–5 μM; Sigma-Aldrich). Cells were lysed in radioimmunoprecipitation assay (RIPA) buffer supplemented with 1.5 mM Na_3_VO_4_, 15 mM NaF, 50 nM calyculin A (Cell Signaling) and a protease inhibitor cocktail (Sigma-Aldrich) and the lysates were cleared by centrifugation at 14.000 x *g* for 60 min at 4°C. Proteins were separated using NuPAGE or Bolt Novex 4–12% gradient Bis-Tris gels (Invitrogen), transferred to Immobilon-P transfer membranes (Millipore Corp), incubated with antibodies and visualized by chemiluminescence (GE Healthcare). Signal intensities were quantified using Image J and normalized to the level of α-tubulin or β4.

### Reverse transcriptase-quantitative polymerase chain reaction

Reverse transcriptase-quantitative polymerase chain reaction (RT-qPCR) analysis was performed in quadruplicate for determination of the levels of PKD and PKC mRNAs in PA-JEB/β4 keratinocytes. Cells were grown in complete KGM medium, or transfected and serum-starved as described above, and lysed in RNA-Bee (Tel-test Inc.) or Trizol^®^ reagent (Invitrogen). Total RNA was separated from DNA and proteins by the addition of chloroform (Sigma-Aldrich) and subsequent centrifugation at 12.000 x *g* for 15 min at 4°C. The RNA was precipitated with isopropanol and washed with 70% ethanol. Integrity of the isolated RNA was assessed by agarose gel electrophoresis.

First strand cDNA synthesis was performed with 3 μg of total RNA using the first strand cDNA synthesis kit K1612 (Thermo Fisher Scientific) following manufacturer’s directions. The PCR reactions were run using SYBR^®^ Advantage^®^ qPCR premix (Clontech) on a 7500 Fast Real-Time PCR system (Applied Biosystems) and the following primers were used: PKD1: forward 5’-GCATCTCGTTCCATCTGCAG-3’, reverse 5’-AGGTAGGGTCATGGCGAAAA-3’; PKD2: forward 5’-AAGTTCCCTGAGTGTGGCTT-3’, reverse 5’-GCAGTGATCACAGAAGGCAG-3’; PKD3: forward 5’-CGCCATGACATGAACTCAGA-3’, reverse 5’-TCCTTGACGTACCAATCCCC- 3’; PKCδ: forward 5’-AGCCGACCATGTATCCTGAG-3’, reverse 5’-ACTGTTTGCAATCCACGTCC-3’; PKCε: forward 5’- CATCGATCTCTCAGGGTCGT-3’, reverse 5’- CCGAAGATAGGTGGCCATGA-3’; PKCη: forward 5’-ACGAGGAGTTTTGCGCTAAC-3’, reverse 5’-ATACTTTCCCCTCTGGCTCG-3’; PKCθ: forward 5’-TTGTCCAACTTTGACTGCGG-3’, reverse 5’-GCATCAAAAGTGCTGTCCCA-3’ and cyclophilin A: forward 5’-CATCTGCACTGCCAAGACTGA-3’, reverse 5’-TTGCCAAACACCACATGCTT-3’.

In the presence or absence of a treatment control, relative mRNA quantities were obtained using the 2^-ΔΔCt^ or 2^-ΔCt^ method, respectively. The ΔCt is the obtained Ct value of PKD or PKC minus the obtained Ct value of cyclophilin A (endogenous control). Graphs were made, and statistical significance was analysed using paired t-tests, in GraphPad Prism 6.

## Results

**Fig 1:**
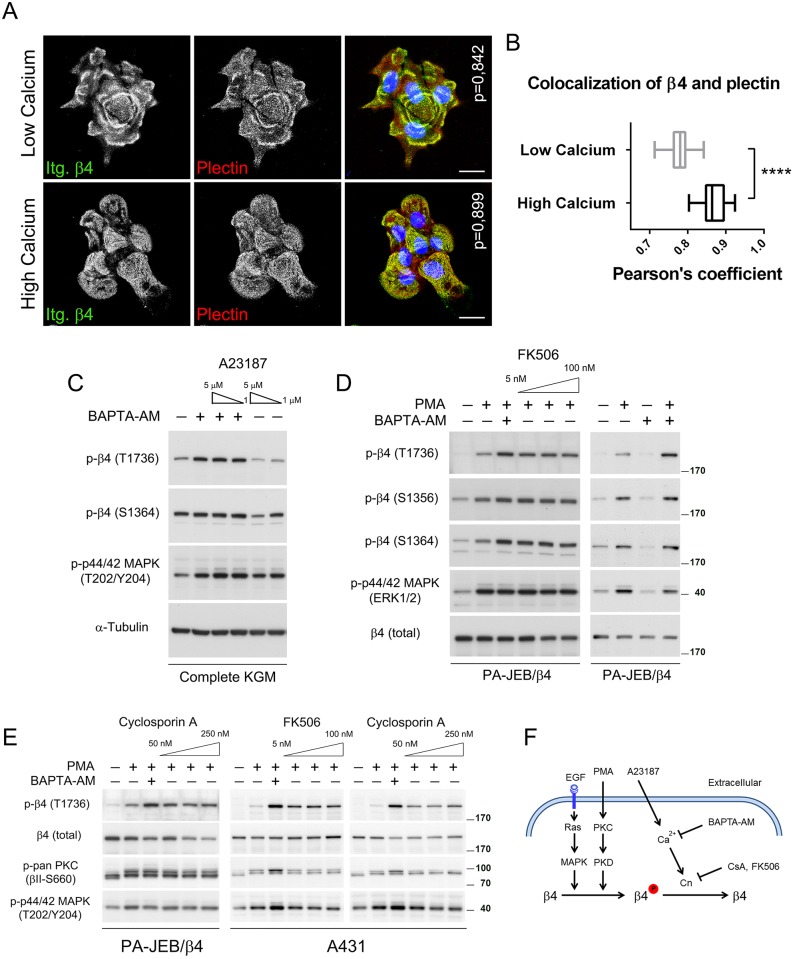
Regulation of hemidesmosome formation and β4-T1736 phosphorylation by extracellular calcium. *A*, Confocal microscopic images show the effect of extracellular calcium on the formation and organization of HDs in PA-JEB/β4 keratinocytes. After 24 hours of growth in either low (KGM; left picture) or high calcium (DMEM; right picture) medium, PA-JEB/β4 keratinocytes were stained with mAbs 439-9B against β4 (green) and 31 against plectin (red). Cells were counterstained with DAPI (bleu) to identify nuclei. HDs are identified as dots containing both β4 and plectin and appear in yellow. Scale bars: 20 μm. *B*, Co-localization between β4 and plectin was quantified for each cell using Pearson’s correlation coefficient. Box plots show median (solid line) and 25th and 75th percentiles (boxes) of the distribution for each condition (n = 3, ~10 images per experiment). **** <0,0001. *C*, PA-JEB/β4 keratinocytes were pre-treated with the intracellular calcium chelator, BAPTA-AM and/or different concentrations of the calcium ionophore A23187 (1–5 μM), or left untreated. Cell lysates were analyzed by immunoblotting with antibodies as indicated. *D*, PA-JEB/β4 keratinocytes were deprived of growth factors overnight, pre-treated with BAPTA-AM, different concentrations (5–100 nM) of FK506 or left untreated before stimulation with PMA. Cell lysates were analyzed by immunoblotting with the indicated antibodies. *E*. PA-JEB/β4 keratinocytes and A431 epidermoid carcinoma cells were serum-starved overnight, pre-treated with BAPTA-AM or different concentrations of FK506 (5–100 nM) or CsA (50–250 nM) and stimulated with PMA or left unstimulated. Cell lysates were analyzed by immunoblotting with the indicated antibodies. *F*. Schematic diagram of the different stimuli and inhibitors along with the signaling pathways that control β4 phosphorylation. Cn, calcineurin; CsA, Cyclosporin A; FK506, tacrolimus; BAPTA-AM, 1,2-*Bis*(2-aminophenoxy)ethane-*N*,*N*,*N*',*N*'-tetraacetic acid tetrakis (acetoxymethyl ester).

In previous studies, we empirically found that keratinocytes form HDs more efficiently when we switched the keratinocyte growth medium from one with a low calcium concentration (KGM) to one with a high calcium concentration (DMEM) 24h before analysis [8]. Here we confirm these findings and show that HDs in the low calcium medium, occur in patches at the cell-substrate contact sites, which often are organized in a typical “cauliflower-like” pattern. In high calcium medium, HDs are more abundant and occupy a large part of the basal surface of cells (Fig 1A and 1B). As the cytosolic calcium levels are affected by the extracellular calcium concentration, and phosphorylation of β4 at S1356 has been associated with a reduced number of HDs [23,24], we studied the effect of the intracellular calcium [Ca^2+^]*i* chelator BAPTA-AM and the calcium ionophore A23187 on the phosphorylation of T1736 and S1364. Treatment of PA-JEB/β4 keratinocytes, grown in complete KGM, with BAPTA-AM resulted in a 2–3 fold increase in the phosphorylation of β4 at T1736. BAPTA-AM did not increase the already high basal level of S1364 phosphorylation, probably because this phosphorylation was already maximal (Fig 1C). In contrast to the BAPTA-AM treatment, A23187 decreased the basal level of phosphorylation of S1364 and T1736. It did not reduce the phosphorylation of these two residues in the presence of BAPTA-AM, likely because the concentration of BAPTA-AM was sufficient to completely prevent the A23187-induced increases in cytosolic calcium ions.

The changes in phosphorylation of T1736 in PA-JEB/β4 keratinocytes treated with BAPTA-AM or A23187 paralleled to some extent those by ERK1/2, which may point to a possible role of the MAPK signaling pathway in the phosphorylation of this residue. However, compared to the phosphorylation of S1364, which is known to be controlled by the MAPK signaling pathway [17], phosphorylation of T1736 may only occur when this pathway is strongly activated.

Because the level of phosphorylation of proteins reflects a balance between protein kinases and phosphatases, we treated PA-JEB/β4 keratinocytes with the calcineurin inhibitor FK506. Consistent with the reported role of calcineurin in the regulation of β4-S1356 phosphorylation [24], we found that the PMA-induced phosphorylation of this residue, as well as that of S1364 was increased by FK506 (Fig 1D). FK506 had no effect on the PMA-induced activation of ERK1/2. A small, but consistent, increase in the PMA-stimulated phosphorylation of β4 was also observed with FK506 at T1736; however, the effect was always less than that seen with BAPTA-AM (Fig 1D). Notably, BAPTA-AM alone did not increase basal phosphorylation of β4 at T1736, S1356 or S1364 (Fig 1D). A small increase in T1736 phosphorylation was also observed when PA-JEB/β4 keratinocytes were treated with the calcineurin inhibitor Cyclosporin A (CsA) (Fig 1E). These effects were not restricted to PA-JEB/β4 keratinocytes, as both FK506 and CsA also slightly increased the PMA-stimulated phosphorylation of T1736 in A431 epidermoid carcinoma cells (Fig 1E).

Together these data indicate that HD formation is regulated by the extracellular calcium concentration, and that phosphorylation of β4 at T1736 is increased by reducing the cytosolic calcium concentration, which may in part be due to the inhibition of calcineurin.

### PMA- and EGF-stimulated phosphorylation of β4 at S1364 and T1736

**Fig 2:**
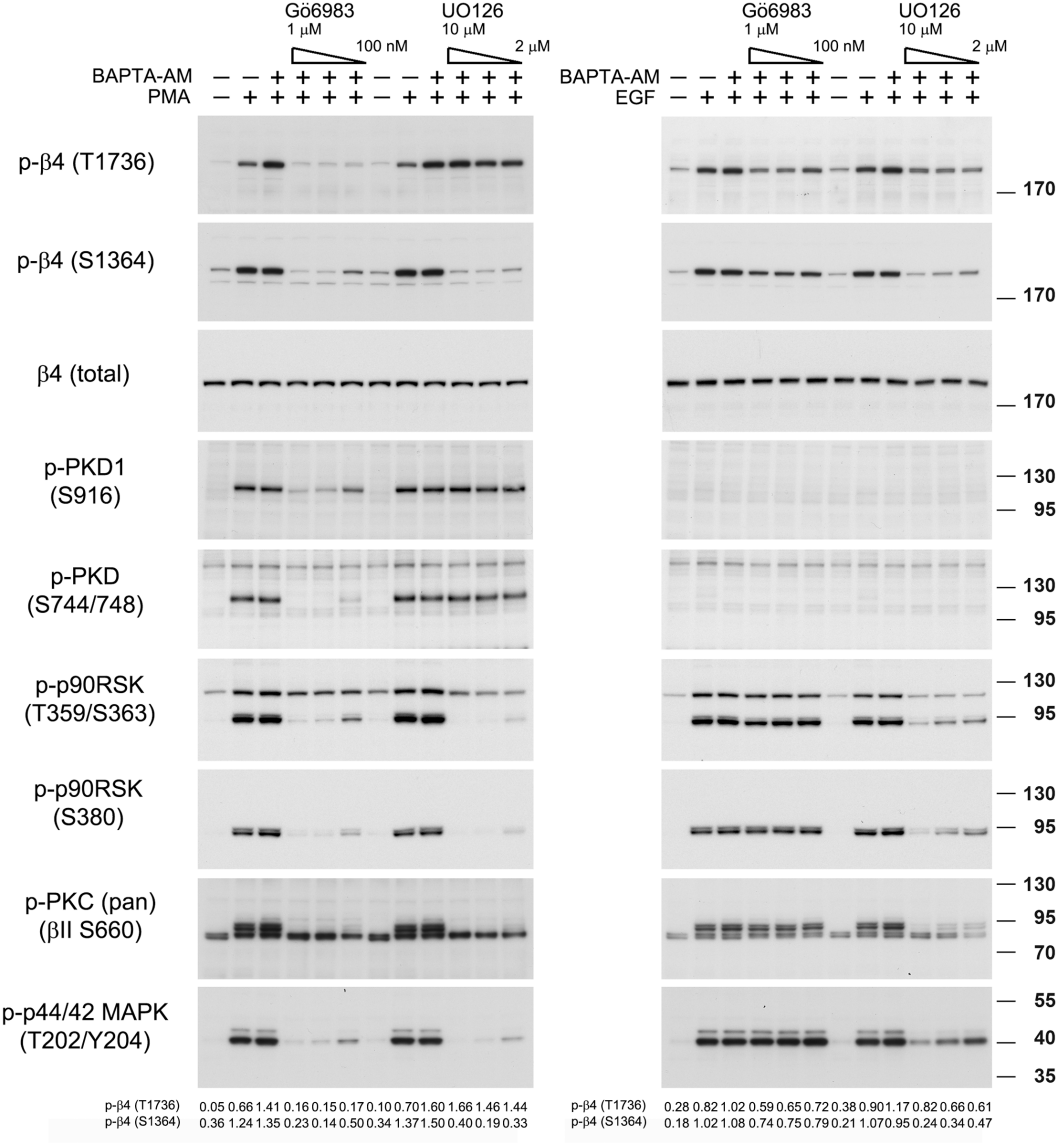
PMA- and EGF-stimulated phosphorylation of β4-T1736 is dependent on PKC and MAPK, respectively. PA-JEB/β4 keratinocytes were deprived of growth factors overnight, pre-treated with BAPTA-AM for 30 min and then treated for 30 min with different concentrations of Gӧ6983 (100 nM-1 μM) or UO126 (2–10 μM). Subsequently the cells were stimulated with EGF or PMA for 10 min. Cell lysates were analyzed by immunoblotting with antibodies as indicated. The values below the blots indicate the signal intensities for the phosphorylated β4-T1736 and β4-S1364 proteins after normalization to the level of β4.

Previously, we have shown that PKD1, a downstream effector kinase of PKC, phosphorylates β4 at T1736 *in vitro*, and that this kinase is activated in PA-JEB/β4 keratinocytes upon stimulation with PMA, but not with EGF [18]. However, the finding that the basal phosphorylation of T1736 is increased by lowering the cytosolic calcium concentrations and is accompanied by an increase in the activity of ERK1/2, suggested that T1736 phosphorylation may also be regulated through MAPK signaling. To gain further insight in the role of PKD and MAPK signaling, we tested the effects of inhibitors of PKC (Gő6983) and MEK1/2 (UO126) on the PMA- and EGF-induced phosphorylation of T1736. Additionally, we investigated the effects of the two inhibitors on the PMA- and EGF-induced phosphorylation of S1364. As shown in Fig 2, while UO126 inhibited both the PMA- and EGF-induced phosphorylation of β4-S1364, Gő6983 only blocked the PMA-stimulated phosphorylation of S1364. These findings are consistent with RSK1/2 being the two kinases that phosphorylate β4-S1364 downstream of PKC and EGFR activation. In contrast, phosphorylation of β4-T1736 by PMA is not sensitive to inhibition by UO126; it is only inhibited by Gő6983 (Fig 2). Since Gő6983, but not UO126, also prevented the PMA-induced phosphorylation of PKD1 at S744/748 (two activation loop sites) by PKC and of S916 (autophosphorylation site), which is correlated with catalytic activity of PKD1, this kinase may indeed be responsible for the phosphorylation of β4-T1736 by PMA. In line with previous results, we found that PKD1 is not obviously activated downstream of the EGFR, and therefore, it is unlikely that this kinase participates in the phosphorylation of T1736 downstream of the EGFR activation [18]. Because the phospho-PKD1-S916 and phospho-PKD1-S744/748 antibodies, used to detect the activation state of PKD1, also recognize the autophosphorylation site in PKD2 and the activation loop sites in PKD2 and PKD3, respectively, a role of these kinases in the EGF-mediated phosphorylation of β4-T1736 can also be excluded.

We again could establish an association between EGF-stimulated phosphorylation of T1736 and MAPK signaling, i.e. the EGF-stimulated phosphorylation of T1736 was reduced by UO126, albeit not completely abolished. We also observed a reduction in the EGF-stimulated phosphorylation of β4-T1736 with Gő6983, but because phosphorylation of PKD1 (PKD2 or PKD3) by PKC could not be demonstrated, the significance of this reduction is not clear. A similar reduction was observed for the phosphorylation of β4 at S1364 and of RSK at T359/S363.

It is perhaps noteworthy that S380 of RSK1/2 is homologous to S660 of PKCβII (both are present in the conserved bulky ring motif) and that the phosphorylation of this residue therefore may also be detected by the phospho-pan PKC antibody. Indeed, the phospho-pan PKC antibody detected phosphorylated proteins that migrated in gel electrophoresis at a position, corresponding to that of phosphorylated RSKs (approximately 90 kDa). Furthermore, the patterns of phosphorylation and sensitivity of these proteins to Gő6983 and UO126 exactly matched those of the phosphorylation of RSK at S380.

In conclusion, the results further suggest a role of RSK1/2 in the phosphorylation of β4 at S1364 and provide further evidence for the role of PKD1 in the phosphorylation of β4 at T1736 downstream of PKC stimulation and of the MAPK signaling downstream of EGFR stimulation.

### PMA-stimulated phosphorylation of β4-T1736 is primarily mediated by PKD2

**Fig 3:**
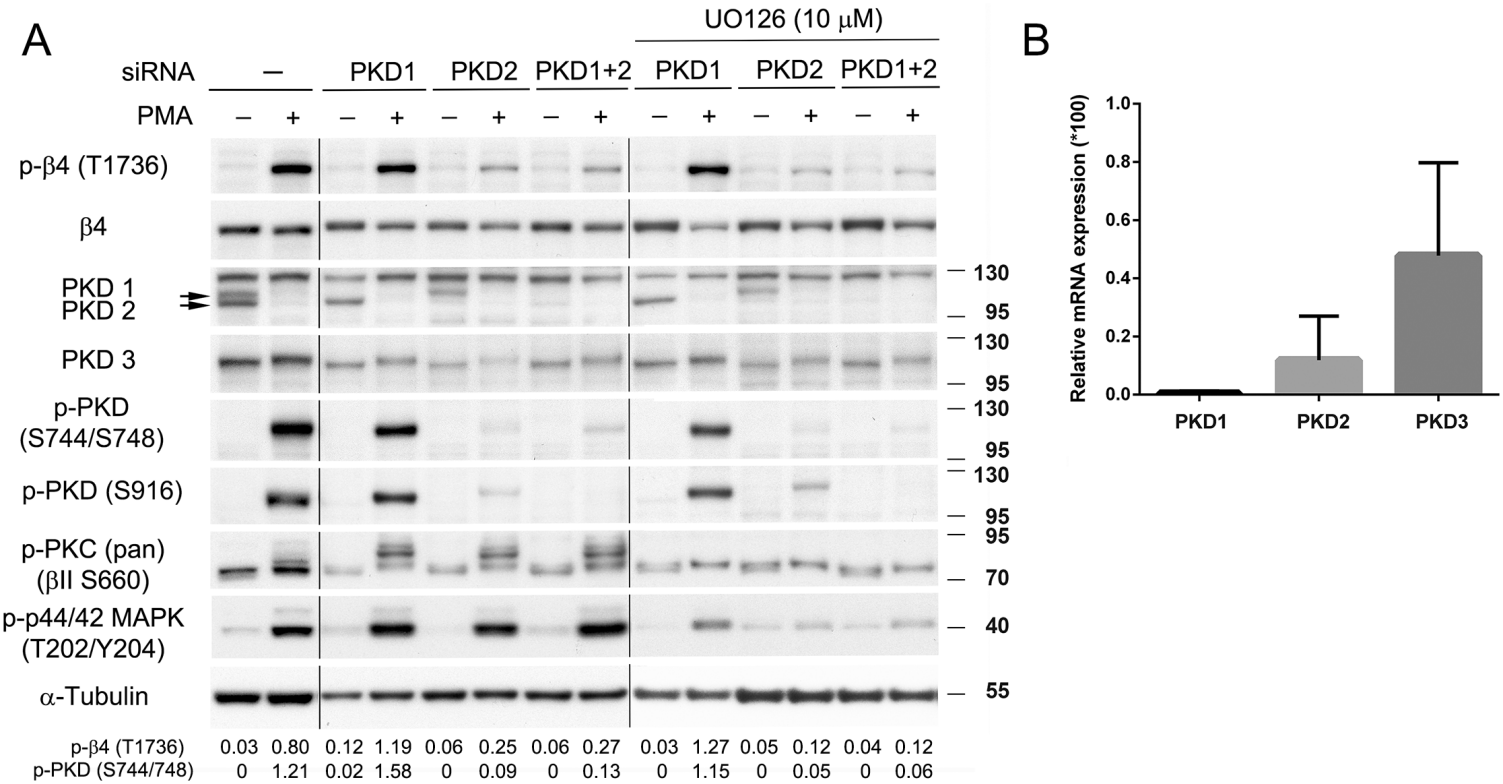
PMA-stimulated phosphorylation of β4-T1736 is primarily mediated by PKD2. *A*. PA-JEB/β4 keratinocytes, not transfected (-) or transfected with siRNAs against PKD1 or PKD2, or a combination of PKD1 and PKD2 siRNAs, were deprived of growth factors overnight, pre-treated with UO126 (10 μM) or left untreated, and then stimulated with PMA for 10 min. The cell lysates were analyzed by immunoblotting with the indicated antibodies. The values below the blots indicate the signal intensities for the phosphorylated β4-T1736 and PKD (S744/748) proteins after normalization to the level of α-tubulin. *B*. Expression levels of the mRNAs for the three PKD family members were determined by RT-qPCR relative to cyclophilin A mRNA levels in PA-JEB/β4 cells. Values are means ± SD from four independent experiments performed in duplo.

To confirm the role of PKD isoforms in PMA-stimulated phosphorylation of T1736, we silenced the expression of endogenous PKD1 and PKD2 in PA-JEB/β4 keratinocytes by transfection with isoform-specific PKD siRNAs. The transfected cells were then treated with PMA to evaluate the contributions of the two PKD isoforms in the phosphorylation of T1736. Fig 3A shows that depletion of PKD2, but not PKD1, strongly reduced the PMA-stimulated phosphorylation of β4 at T1736. The residual phosphorylation of T1736, remaining after depletion of PKD2, could not be attributed to PKD1, because the combined depletion of PKD1 and PKD2 did not further decrease T1736 phosphorylation. The efficiency of the siRNA-mediated depletion of both PKD1 and PKD2 was confirmed, not only by the failure of the anti-PKD1/2 antibody to react with unstimulated cells, but also by the absence of reactivity of the phospho-PKD1-S916 antibody with the PMA stimulated cells. Noticeably, because the anti-PKD1/2 antibody has been generated against the synthetic peptide corresponding to residues surrounding S916, it does not recognize these two kinases when phosphorylated at S916.

Furthermore, a role of the MAPK signaling cascade in the remaining phosphorylation of T1736 could be excluded. No further reduction in the PMA-stimulated phosphorylation of T1736 was observed in UO126-treated cells depleted of PKD1, PKD2 or both proteins. Next, we considered the possibility that the residual phosphorylation of β4 at T1736 is caused by the activation of PKD3. RT-qPCR showed that PKD3 is indeed expressed by PA-JEB/β4 keratinocytes (Fig 3B). In fact, its expression is much stronger than that of PKD1 and PKD2. The PKD3 protein could also be detected by antibodies on immunoblots and showed a mobility shift upon PMA stimulation, indicative of phosphorylation and activation of the kinase. Consistent with the activation of PKD3 by PMA, depletion of both PKD1 and PKD2 did not completely abolish the reactivity of the phospho-PKD1-S744/748 antibody. Some residual reactivity remained detectable.

Together these results indicate that the PMA induced phosphorylation of β4 at T1736 is primarily mediated by PKD2 with a possible small contribution by PKD3.

### PMA-induced PKD activation is mediated by PKCδ

**Fig 4:**
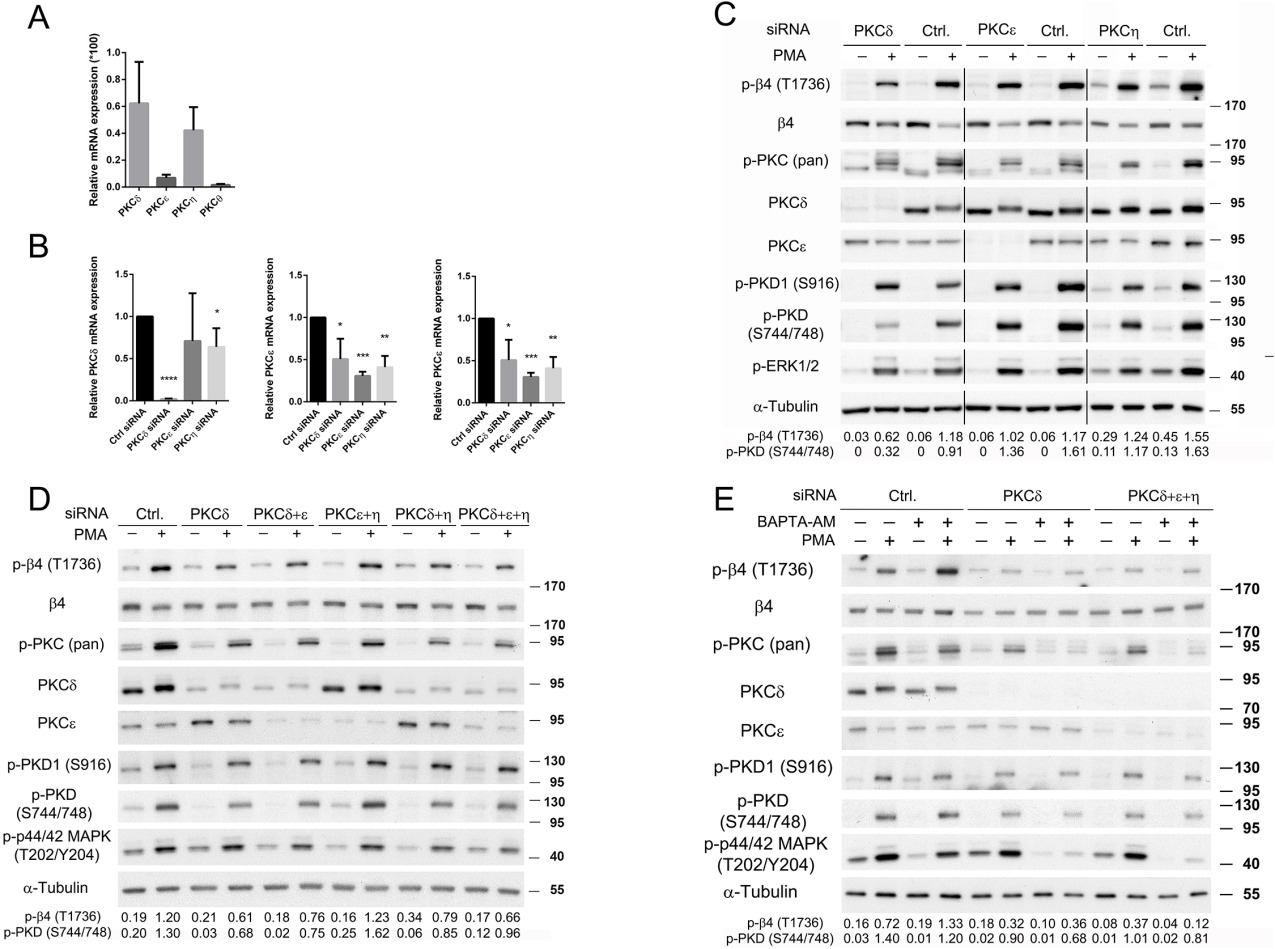
PKCδ mediates PMA-induced PKD activation. *A*. Expression levels of the indicated mRNAs for the four novel PKCs were determined by RT-qPCR and normalized to cyclophilin A mRNA levels in PA-JEB/β4 cells. Values are means ± SD from four independent experiments performed in duplo. *B*. Relative mRNA levels of PKCδ, PKCε and PKCη were determined by RT-qPCR in PA-JEB/β4 cells, transfected with control (Ctrl) siRNA or siRNAs targeting PKCε, PKCδ, or PKCη. The values were normalized to cyclophilin A mRNA and compared to the mRNA levels in the cells treated with control siRNA. They are the means ± SD from four independent experiments performed in duplo. ****P, <0.0001; ***P, <0.001; **P, <0,01; *P, <0,05. *C*, *D*. PA-JEB/β4 keratinocytes were transfected with single siRNAs or combinations of siRNAs as indicated. After 15 h, the cells were deprived of growth factors for 24h and subsequently stimulated with PMA for 10 min or left unstimulated. Cell lysates were analyzed by immunoblotting with antibodies as indicated. *E*. PA-JEB/β4 keratinocytes, transfected with control, PKCδ or PKCδ siRNA in combination with PKCε and PKCη siRNAs, were deprived of growth factors for 24h, pre-treated with BAPTA-AM for 1h and subsequently treated with PMA for 10 min or left untreated and stimulated. Cell lysates were analyzed by immunoblotting with antibodies as indicated. The values below the blots shown in panels C, D and E indicate the signal intensities for the phosphorylated β4-T1736 and PKD (S744/748) proteins after normalization to the level of α-tubulin.

Previous studies have implicated Ca^2+^-independent, “novel” PKCs as major upstream kinases in the phosphorylation of the PKD activation loop [25]. PA-JEB/β4 keratinocytes express three of the four novel PKCs, including PKCε, PKCδ and PKCη, as judged by RT-qPCR (Fig 4A). The expression of PKCε is relatively low compared to that of PKCδ and PKCη. To test which of these PKCs mediates phosphorylation of the PKD activation loop, we used specific PKC siRNAs to suppress their expression individually and in any of the possible combinations of the proteins, two-and-two and all three together. The knockdown efficiency for each PKC isoform was confirmed by RT-qPCR and/or immunoblot analysis (Fig 4B and 4C).

Both the PMA-induced PKD activation loop phosphorylation at S744/748 and the phosphorylation of β4 at T1736 are attenuated when PKCδ expression is suppressed (Fig 4C). However, depletion of PKCδ seems to have only little effect on the activity of PKD1/2, as measured by the phospho-PKD-S916 antibody. On the contrary, phosphorylation of either β4 or PKD was not obviously reduced following depletion of PKCε or PKCη (Fig 4C), or when these two PKC isoforms were depleted together (Fig 4D). Furthermore, combining siRNA-mediated depletion of PKCδ with either PKCε or PKCη, or with both PKCε and PKCη did not result in a further reduction of the phosphorylation of β4-T1736 or of the activation loop phosphorylation of PKD other than that already seen after depletion of PKCδ alone (Fig 4D).

Although depletion of PKCδ substantially reduced the PMA-induced β4-T1736 phosphorylation, it did not completely abrogate it. This may be due to incomplete siRNA-mediated depletion of PKCδ, or to the existence of an alternative kinase that phosphorylates PKD-S744/748 and β4-T1736. The possibility that PKCδ cooperates with “classical” Ca^2+^-dependent PKCs in mediating PMA-induced phosphorylation of PKD-S744/748 and β4-T1736 could be excluded, because the combination of siRNA-mediated depletion of PKCδ with BAPTA-AM did not reduce the PMA-induced phosphorylation of β4-T1736 further than that already caused by siRNA-mediated depletion of PKCδ alone (Fig 4E). Even after depleting all three PKC isoforms, BAPTA-AM treatment did not completely abrogate the PMA-stimulated phosphorylation of β4-T1736. The fact that PKCδ depletion reduced the PMA-induced phosphorylation of β4-T1736 to a similar extent in both BAPTA-AM treated and untreated cells indicates that the effects of BAPTA-AM on the PMA-induced phosphorylation of β4-T1736 are mediated through PKCδ.

We conclude that efficient PMA-induced phosphorylation of β4-T1736 is dependent on PKCδ but not PKCε or PKCη, or the “classical” PKCs. The remaining phosphorylation of PKD (S744/748 and S916) and β4-T1736 seen after combined treatment with PKCδ siRNA and BAPTA-AM treatment may indicate that some phosphorylation of PKD and β4 is PKCδ independent.

### EGF-dependent phosphorylation of β4 depends on RSK1/2

**Fig 5:**
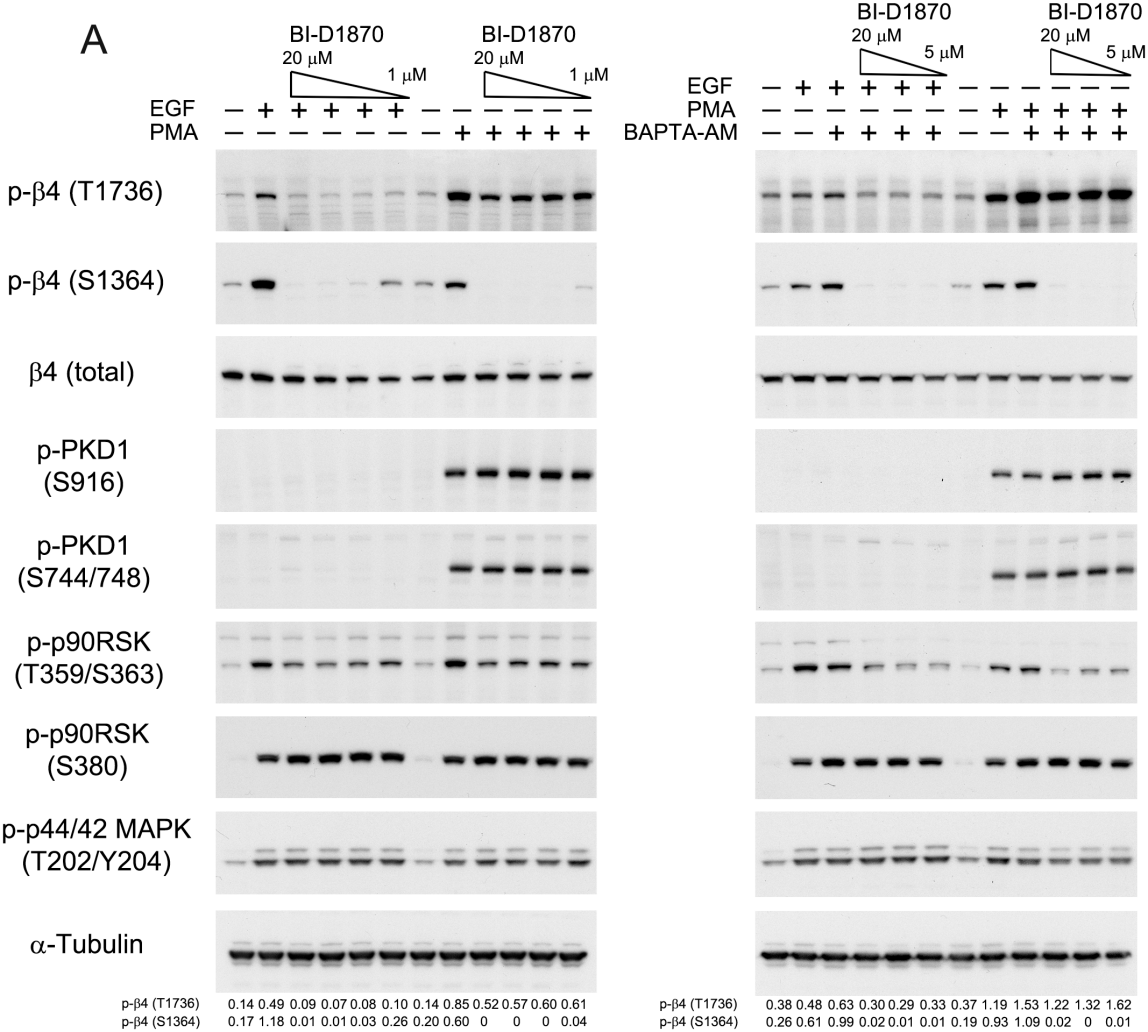
EGF- but not PMA-stimulated phosphorylation of β4 at T1736 and S1364 depends on RSK1/2. PA-JEB/β4 keratinocytes were deprived of growth factors overnight, pre-treated with BAPTA-AM for 30 min and/or BI-D1870 for 30 min at the indicated concentrations or left untreated and then stimulated with EGF or PMA. The cell lysates were analysed by immunoblotting with the indicated antibodies. The values below the blots indicate the signal intensities for the phosphorylated β4-T1736 and β4-S1364 proteins after normalization to the level of α-tubulin.

The results obtained so far showed that EGF-stimulated phosphorylation of β4-T1736 is not mediated by PKD, but is dependent on ERK1/2 signaling. To investigate whether RSK, a known downstream effector kinase of ERK1/2, is involved in EGF-stimulated phosphorylation of β4 at T1736, we pre-treated PA-JEB/β4 keratinocytes with the RSK inhibitor BI-D1870 and subsequently stimulated the cells with EGF or PMA in the presence or absence of BAPTA-AM. BI-D1870 acts as an ATP-competitive inhibitor of the N-terminal AGC kinase domain of RSK, but does not inhibit the C-terminal kinase, whose activity is regulated by ERK1/2 [26]. As shown in Fig 5, inhibition of RSK by BI-D1870 reduced the EGF-stimulated phosphorylation of β4-T1736 to basal levels. BI-D1870 also slightly inhibited the PMA-induced (PKD2-mediated) phosphorylation of T1736, while it had no inhibitory effect on the activity of PKD, as judged by the phosphorylation of PKD2 at S916 and S744/748. Pre-treatment with BAPTA-AM did not alter the inhibitory effect of BI-D1870 on the PMA- and EGF-stimulated phosphorylation of T1736. Consistent with our previous results, BI-D1870 completely abolished the phosphorylation of S1364 induced by PMA or EGF [17]. Unexpectedly, we found that BI-D1870 exerted a small, but consistent, inhibitory effect on the ERK1/2-dependent phosphorylation of RSK at T359/S363, while it had no effect on the activity of ERK1/2, or on the autophosphorylation of RSK at S380 (mediated by its C-terminal kinase domain). Together these data suggest that downstream of ERK1/2, RSK plays an important role in the EGF-stimulated phosphorylation of β4 at T1736.

### Kinase-inactive, but not wild-type, RSK1 or RSK2 reduces β4-T1736 phosphorylation

**Fig 6:**
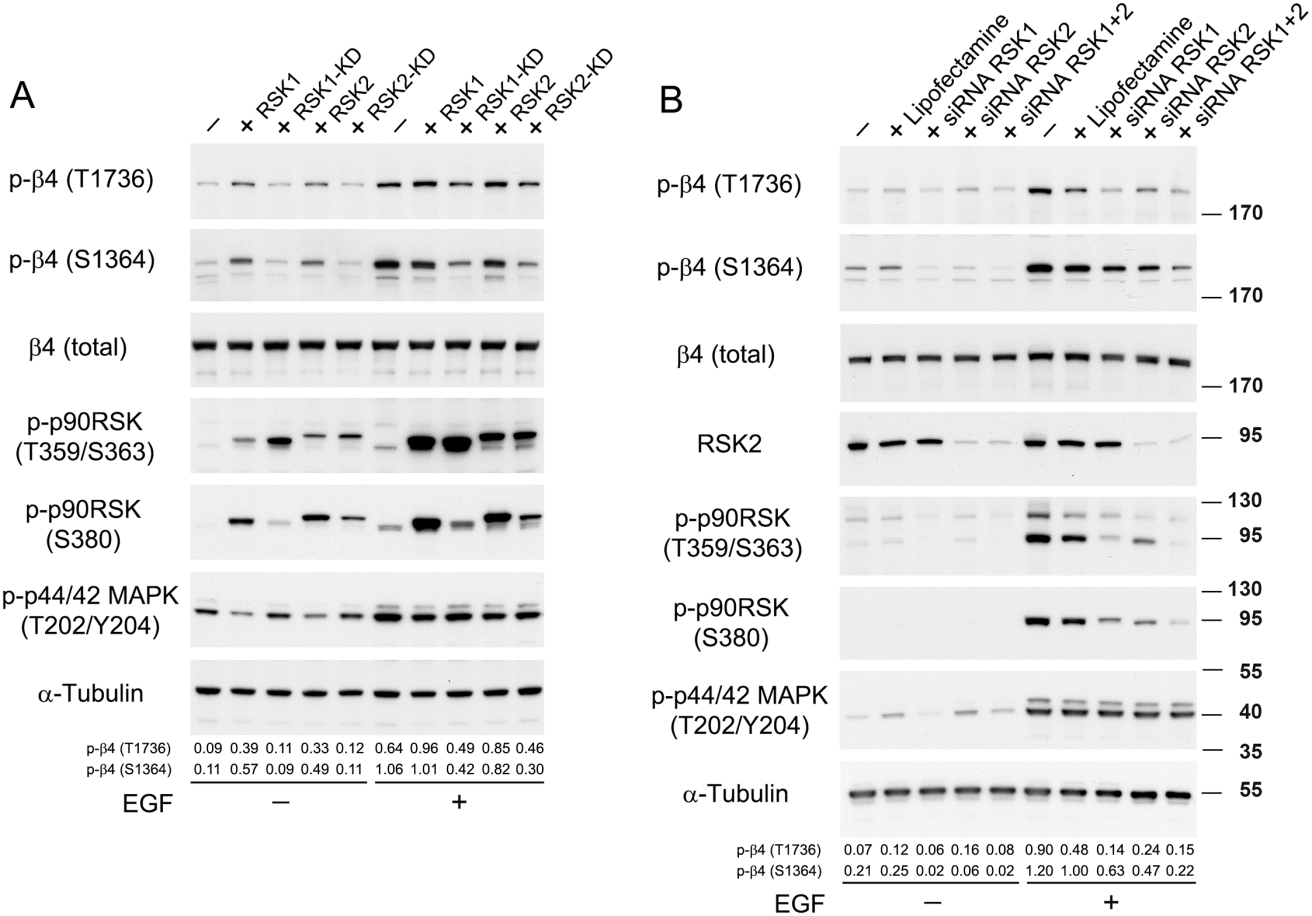
EGF-stimulated phosphorylation of β4 at T1736 is mediated by RSK1. *A*. PA-JEB/β4 keratinocytes, transiently overexpressing RSK1, RSK2, kinase-dead RSK1 or kinase-dead RSK2, were deprived of growth factors overnight and stimulated with EGF or left unstimulated. The cell lysates were analysed by immunoblotting with antibodies as indicated. *B*. PA-JEB/β4 keratinocytes transfected with RSK1 and/or RSK2 siRNAs were deprived of growth factors overnight and stimulated with EGF or left unstimulated. The cell lysates were analysed by immunoblotting with the indicated antibodies. The values below the blots indicate the signal intensities for the phosphorylated β4-T1736 and β4-S1364 proteins after normalization to the level of α-tubulin.

Next, we overexpressed wild-type RSK1 and RSK2, and kinase-inactive mutants thereof in PA-JEB/β4 keratinocytes, and compared the phosphorylation of β4 at T1736 with that at S1364. Overexpression of wild-type RSK1 or RSK2 increased the phosphorylation of β4-T1736 and β4-S1364 in unstimulated keratinocytes, but not (S1364) or only slightly (T1736) in keratinocytes stimulated with EGF, probably because these residues are already (almost) maximally phosphorylated by the endogenous kinases (Fig 6A). The increased phosphorylation of β4-T1736 and β4-S1364 is correlated with elevated levels of phosphorylation of RSK at S380, T359 and S363, which is in accordance with results of the inhibition studies with BI-D1870 that phosphorylation of β4 at T1736 and S1364 requires active RSK. Overexpression of kinase-inactive RSK1 and RSK2, on the other hand, did not lower the basal level of phosphorylation of β4 at T1736 or S1364 in unstimulated cells, but reduced the EGF-stimulated phosphorylation of these residues, albeit that of S1364 more substantially than that of T1736 (Fig 6A). Interestingly, we found that the two RSK isoforms have a feedback inhibitory effect on the activity of ERK1/2. The level of both phosphorylated ERK1/2 (T202/Y204) and phosphorylated RSK (T359/S363) diminished in the cells overexpressing wild-type RSK1 or RSK2, as compared to in those overexpressing the kinase-inactive mutants of RSK1 or RSK2 under basal conditions. Although we anticipated that phosphorylation of the two kinase-inactive RSK mutants at S380 would be completely absent, low levels of phosphorylation could still be observed. This particularly in the case of the RSK2 mutant, suggesting that this mutant is not completely kinase-dead. The remaining activity of this “kinase-inactive” mutant, however, is not sufficient for inducing phosphorylation of β4-T1736 and β4-S1364 in unstimulated keratinocytes.

In summary, the results lend further support to the contention that like β4-S1364, T1736 is a target for RSK-mediated phosphorylation.

### Depletion of RSK1, but not RSK2, reduces β4 T1736 phosphorylation

We next investigated the effects of depletion of RSK1 and RSK2 on the phosphorylation of β4-T1736 by siRNA-mediated knockdown (Fig 6B). A small but reproducible reduction in the basal level of phosphorylation of β4-T1736 was observed when PA-JEB/β4 keratinocytes were transfected with a siRNA against RSK1, but not with one against RSK2. Moreover, simultaneous depletion of RSK1 and RSK2 did not reduce the basal level of β4-T1736 phosphorylation to a greater extent than depletion of RSK1 alone. Comparable effects of RSK1 and RSK2 depletion were obtained for β4-S1364 phosphorylation, except for a small reduction in β4-S1364 phosphorylation by RSK2 depletion. Overall, the effects of RSK1 and RSK2 depletion on the phosphorylation of the two β4 residues closely correspond to the activity of these kinases as measured by the levels of phosphorylated RSK at T359/S363 and S380. Interestingly, depletion of RSK1 either alone or together with RSK2 caused a reduction in the basal activity of ERK1/2. Since a similar reduction was seen after overexpression of wild-type RSK1 or RSK2 in PA-JEB/β4 keratinocytes (Fig 6A), it further supports the notion that the expression of RSK and the activity of ERK1/2 are intimately connected.

While the treatment of cells with the lipid-based transfection reagent had no effect on the basal levels of β4-T1736 phosphorylation, it caused a substantial reduction in the EGF-stimulated phosphorylation of β4-T1736. However, a further reduction was seen when also RSK1 expression was depleted. In contrast, depletion of RSK2 had no or only little effect on the EGF-stimulated phosphorylation of β4-T1736, when compared with that of the transfection reagent control. Furthermore, simultaneous depletion of RSK1 and RSK2 did not result in a greater reduction in the phosphorylation of β4-T1736 than is seen after depletion of RSK1 alone, indicating that the RSK2 activity remaining after RSK1 depletion was insufficient to induce phosphorylation of β4-T1736. In contrast, depletion of RSK2 caused a substantial reduction in the EGF-stimulated phosphorylation of β4-S1364 comparable to that of RSK1 depletion. Depletion of both RSK1 and RSK2 reduced the phosphorylation of β4-S1364 to almost basal levels. Thus, while the EGF-stimulated activation of RSK2 is insufficient to induce phosphorylation of T1736, it induces that of S1364. We conclude that both RSK1 and RSK2 are required for the basal and EGF-stimulated phosphorylation of β4-S1364, while RSK1 is mainly responsible for the phosphorylation of β4 at T1736.

### PI3K/Akt signaling pathway is not required for EGF-stimulated phosphorylation of β4

**Fig 7:**
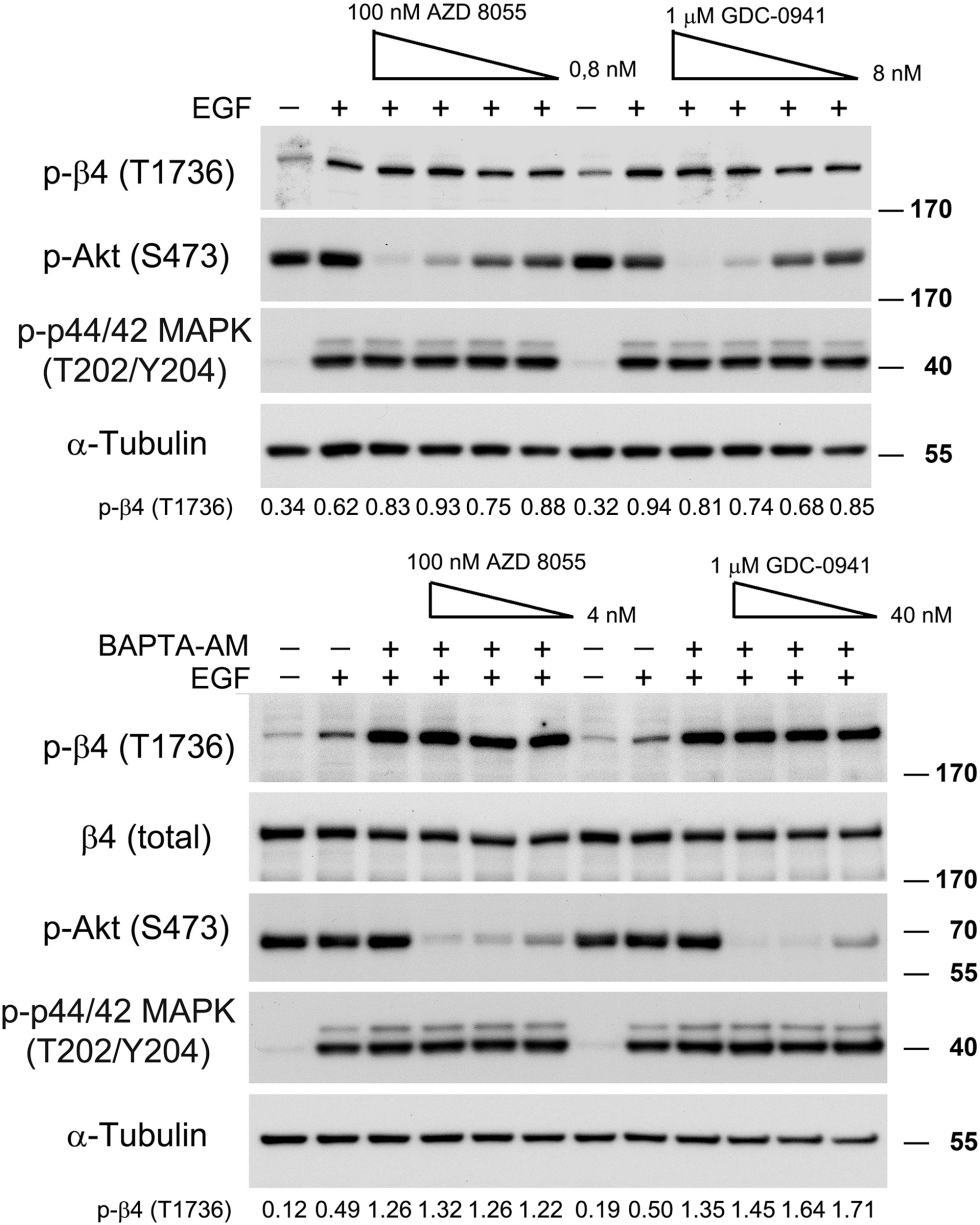
PI3K and mTOR are not required for EGF-mediated β4 phosphorylation. PA-JEB/β4 keratinocytes were deprived of growth factors overnight, pre-treated with BAPTA-AM and/or different concentrations of the mTOR kinase inhibitor AZD 8055 (0,8–100 nM) or the PI3K inhibitor GDC-0941 (8 nM- 1 μM) and subsequently stimulated with EGF or left unstimulated. The cell lysates were analysed by immunoblotting with the indicated antibodies. The values below the blots indicate the signal intensities for the phosphorylated β4-T1736 after normalization to the level of α-tubulin.

Although our findings clearly show that the MAPK signaling pathway is involved in the EGF-stimulated phosphorylation of β4 at T1736, they do not exclude the possibility that additional (EGFR activated) pathways also play a role in this phosphorylation event. One major signaling pathway that relies on EGFR is the PI3K/Akt signaling pathway. This pathway plays a crucial role in cell growth and survival. We found that the PI3K/Akt signaling cascade is constitutively activated in PA-JEB/β4 keratinocytes, as indicated by the phosphorylation of Akt on S473 and T308 (Fig 7, not shown). To investigate whether phosphorylation of β4 is dependent on sustained Akt activation, PA-JEB/β4 keratinocytes were treated with the PI3K inhibitor (GDC-0941) and the mTOR kinase inhibitor (AZD 8055). As shown in Fig 7, stimulation of the PA-JEB/β4 keratinocytes with EGF led to an increase in the phosphorylation of β4 at T1736, which occurred irrespective of whether the cells had been treated with GDC-0941 or AZD 8055. Likewise, the two inhibitors did not antagonize the EGF-stimulated phosphorylation of T1736, which is further increased by the exposure of the keratinocytes to BAPTA-AM. These results indicate that sustained activation of the PI3K/Akt signaling pathway in PA-JEB/β4 is not required for phosphorylation of β4 at T1736.

### MAPK inhibition prevents EGF-induced HD dissolution

**Fig 8:**
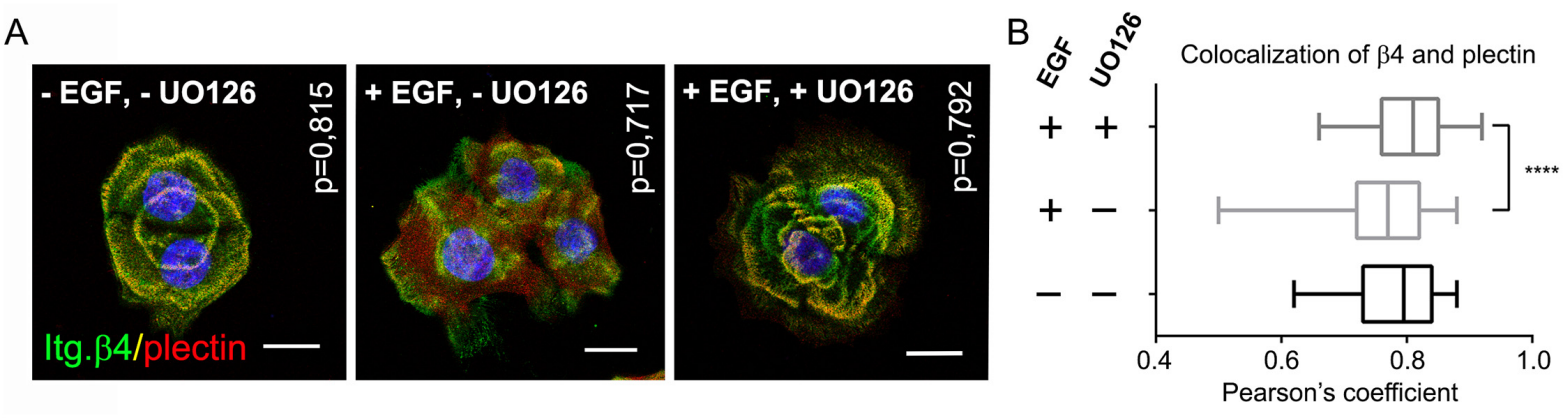
EGF-induced HD dissolution is dependent on MAPK signaling. PA-JEB/β4 keratinocytes, seeded on coverslips, were deprived of growth factors overnight, pre-treated with the MEK inhibitor UO126 and/or stimulated with EGF. *A*. PA-JEB/β4 keratinocytes were stained with mAbs 439-9B against β4 (green) and 31 against plectin (red). Cells were counterstained with DAPI (blue). Co-localization of β4 and plectin in HDs appears in yellow. Scale bars: 20 μm. *B*. Co-localization between β4 and plectin was quantified using Pearson’s correlation coefficient for each image. Box plots show median (solid line) and 25th and 75th percentiles (boxes) of the distribution for each condition (n = 4, ~20 images per experiment). ****P, <0.0001.

We next sought to establish the importance of the EGF-stimulated MAPK signaling pathway in the dissolution of HDs by determining the co-localization between plectin and integrin β4 in PA-JEB/β4 keratinocytes treated with EGF in the presence or absence of the MEK inhibitor UO126. As shown in Fig 8, pre-treatment of PA-JEB/β4 keratinocytes with UO126 prevented the EGF-induced disassembly of HDs. Treated and untreated PA-JEB/β4 keratinocytes showed similar “cauliflower-like” patterns of HDs, while in the EGF-treated cells, not pre-treated with UO126, HDs appeared more diffuse and their number was reduced. We conclude that inhibition of the MAPK signaling pathway prevents EGF-induced HD dissociation.

## Discussion

The interaction between integrin α6β4 and plectin, that is critical for the formation of HDs is dynamically regulated by phosphorylation of β4 at several residues. Two of these residues, S1356 and S1364, are located in the connecting segment of the β4 cytoplasmic domain and their phosphorylation by ERK1/2 and RSK1/2 prevents interaction of the ABD of plectin with β4 [17]. The phosphorylation of a third residue, T1736, located in the C-terminal end of the β4 cytoplasmic domain causes dissociation of β4 from the plakin domain, which is located next to the ABD of plectin [18]. In a previous study, we presented evidence suggesting that T1736 is directly phosphorylated by PKD1 *in vitro* [18]. In this study, we show that, although PA-JEB/β4 keratinocytes express all three PKD isoforms, PMA-induced phosphorylation of β4-T1736 is primarily mediated by PKD2. RT-qPCR analysis revealed that in PA-JEB/β4 keratinocytes the RNA levels of the three PKD isoforms differ greatly, with PKD3 being expressed at a much higher level than PKD2, while PKD1 is expressed at a relatively low level. The low level of PKD1 expression is not obvious from the reactivity of the anti-PKD antibody on Western blots. On these blots, the two PKD isoforms appear to be expressed at comparable levels. The discrepancy between the RNA and protein data may be explained by a different affinity of the antibody for the two PKD isoforms, i.e. the anti-PKD antibody may react more strongly with PKD1 than with PKD2. Interestingly, we found that despite the strong expression of PKD3 at the transcriptional level, the involvement of PKD3 in the phosphorylation of β4-T1736 is negligible. Why PKD3 is unable to efficiently phosphorylate β4-T1736 is not known. It may be due to a different subcellular localization of PKD3 and β4.

PKD activation by PMA in cells is mediated by “novel” Ca^2+^-independent PKCs through phosphorylation of S744/748 in the activation loop [24]. PA-JEB/β4 keratinocytes express three of the four novel PKCs, PKCɛ, PKCη and PKCδ, but only the latter seems to participate in the PKD-mediated phosphorylation of β4-T1736. PKC isoform-specific activation of PKD has been previously shown by Storz et al. [27]. These authors showed that in response to oxidative stress, PKCδ but not PKCɛ mediates PKD activation. Importantly, PKCɛ-mediated phosphorylation of PKD has been demonstrated in co-transfection experiments of PKD with an active form of PKCɛ [27–29]. Thus, the inability of PKCɛ to activate PKD cannot be simply attributed to an inability of the kinase to recognize its substrate. Rather, it seems that PKCɛ does not contribute significantly to PKD activation because of its low expression in PA-JEB/β4 keratinocytes.

There is evidence that PKCδ, but not PKCɛ or PKCη can directly phosphorylate β4 and downregulate its expression [30]. However, PKCδ does not mediate this effect through direct phosphorylation of β4-T1736, because depletion of PKD2, one of the downstream effector kinases of PKCδ, almost completely prevented the PMA-induced phosphorylation of β4-T1736.

In contrast to the PKD2-mediated phosphorylation of β4-T1736 after stimulation with PMA, the EGF-induced phosphorylation of this residue is not mediated by PKD2, but is under the control of the Ras-MAPK signaling pathway. We found that pharmacological inhibition of MEK1/2 or RSK using the inhibitor UO126 or the pan RSK inhibitor BI-D1870, respectively, reduced the EGF-induced phosphorylation of β4-T1736. Moreover, overexpression of RSK1 or RSK2 enhances phosphorylation of β4-T1736, whereas expression of kinase inactive RSK1 and RSK2 antagonizes this phosphorylation. Although these data suggest that both RSK1 and RSK2 can mediate phosphorylation of β4-T1736, studies with RSK1 and RSK2 RNA interference revealed that in PA-JEB/β4 keratinocytes phosphorylation of β4-T1736 is largely dependent on RSK1. This is in contrast to the phosphorylation of β4-S1364 that is dependent on both RSK1 and RSK2. Since no other effector kinases downstream of RSKs have been identified [31], we assume that RSK1 and RSK2 are the kinases that also directly phosphorylate β4-S1364, while RSK1 phosphorylates β4-T1736. In support of this assumption, all four RSKs have been identified as major candidate kinases that can phosphorylate S1364 and T1736 using the Kinexus PhosphoNet kinase predictor (http://www.phosphonet.ca/kinasepredictor). MNK1/2, like the RSKs, are downstream effector kinases of ERK1/2 and have not been identified as candidate protein kinases for the phosphorylation of β4-T1736 by the predictor. Correspondingly, we have not observed a reduction in EGF-stimulated phosphorylation of β4-T1736 with the MNK1/2 inhibitor CPG 57380 (unpublished data). In addition, we demonstrated that the PI3K pathway does not contribute to the phosphorylation of β4-T1736.

**Fig 9:**
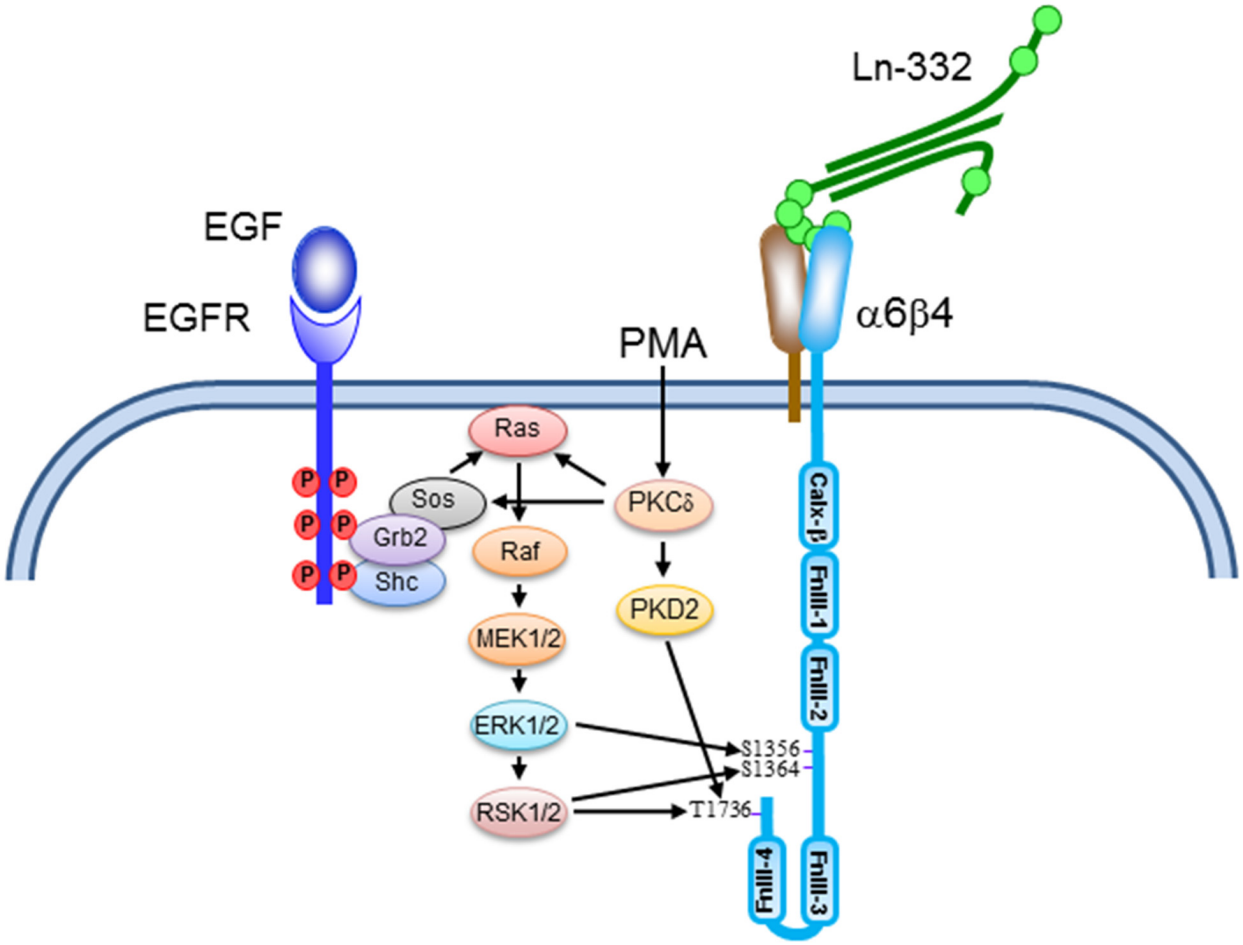
Signaling pathways controling β4 phosphorylation at serine and threonine residues.

Although the RSK1 depletion and inhibition studies with BI-D1870 clearly implicate RSK1 as the sole kinase downstream of EGFR activation that phosphorylates T1736, the finding that phosphorylation of β4-T1736 is not completely inhibited by UO126 suggests that (an)other kinase(s) acting upstream of MEK1/2 may also play a role. Raf kinases which act upstream of MEK1/2 can be excluded as a candidate kinase because BI-D1870, the pan RSK inhibitor, that acts downstream MEK1/2 in the MAPK pathway, completely prevented EGF-stimulated phosphorylation of β4-T1736. It has been reported that UO126 may have other pharmacological effects independently of MEK inhibition, including the activation of protein kinases [32,33]. It is therefore possible that the phosphorylation of β4-T1736, remaining after MEK inhibition, is due to the activation of other kinases and is not part of the signaling pathways downstream of EGFR. We also observed a small reduction in the EGF-stimulated phosphorylation of β4-T1736 with Gő6983. However, because a similar reduction was seen of β4-S1364, we assume that this is due to non-specific inhibition of RSK by the PKC inhibitor Gő6983. A diagram summarizing the signaling pathways leading to β4 phosphorylation downstream of PKC and EGFR stimulation is presented in Fig 9.

Calcineurin is a serine/threonine phosphatase that is activated by the calcium/calmodulin complex [34]. Recent studies have implicated this phosphatase in the regulation of phosphorylation of β4-S1356 through a MAPK-dependent pathway [24]. In line with this study, we show that in the presence of the calcineurin inhibitors CsA and FK506 the PMA-stimulated phosphorylation of β4 was increased not only on S1356, but also on S1364 and T1736. However, the effects were relatively small and neither CsA nor FK506 increased the PMA-induced activation of ERK1/2. On the contrary, BAPTA-AM markedly increased the PMA-stimulated phosphorylation of β4-T1736. In A431 cells, but not in PA-JEB/β4-keratinocytes, this increased phosphorylation was paralleled by an increase in ERK1/2 activation (Fig 1D). In a previous study, it has been shown that chelation of [Ca^2+^]*i* with BAPTA-AM enhances and prolongs EGF-induced ERK1/2 activation in mouse embryo fibroblasts [35]. However, exposure of cells to BAPTA-AM in the absence of EGF had no effect on the activity of ERK1/2 [35]. We confirm these results in PA-JEB/β4 cells and additionally found that FK506 and CsA are also unable to induce ERK1/2 activation in the absence of EGF or PMA (unpublished data). As it has been suggested that the negative role of [Ca^2+^]*i* on the activation of ERK1/2 is caused by the induction of Ca^2+^-dependent expression of MAP kinase phosphatases [35], the applied period of EGF stimulation (10–15 min) might simply be too short to induce the expression of these phosphatases in PA-JEB/β4 keratinocytes. How calcineurin decreases β4 phosphorylation at T1736 is not known, but it may involve the activation of one or more phosphatases that directly dephosphorylate this residue. In this regard, it is perhaps worth mentioning that we have previously shown that treatment of cells with calyculin A, a potent protein phosphatase 2A (PP2A) inhibitor, strongly augments β4-T1736 phosphorylation [18,36].

Our finding, that RSK regulates the interaction between β4 and plectin via phosphorylation of T1736, combined with previous observations that ERK1/2 and RSK regulate the interaction between β4 and the ABD of plectin, implicate the ERK/MAPK signaling pathway as a crucial signaling cascade in keratinocyte migration through controlling HD stability. Indeed, inhibition of the MAPK pathway by UO126 prevents EGF-induced HD dissolution. Recent evidence suggests that HD stability may also be controlled by the ERK-MNK2 pathway through the phosphorylation of plectin at S4642 [37]. This phosphorylation attenuates the interaction of plectin with keratin filaments, and may lead to the destabilization of HDs, as supported by a study showing that in keratinocytes devoid of all keratins plectin is not localized in HDs [38]. In addition to a role of MAPK signaling as an upstream regulator of HD stability and keratinocyte migration, there is data suggesting that MAPK signaling can also be activated downstream of α6β4 and promote keratinocyte migration [39]. However, this activity of α6β4 has been linked to the binding of Shc to tyrosine phosphorylated β4 and may occur only in transformed keratinocytes, not in normal immortalized keratinocytes [40,41]. Indeed, we have found no evidence that β4 is tyrosine phosphorylated in PA-JEB/β4 keratinocytes upon EGF stimulation (unpublished data).

In conclusion, we provide evidence, that depending on the stimulus, PKD2 or RSK1 mediates phosphorylation of β4-T1736, a site that regulates the interaction of β4 with the plakin domain of plectin.
